# The prevalence and determinants of health anxiety during the covid-19 pandemic: A systematic review and meta-analysis

**DOI:** 10.1371/journal.pmen.0000120

**Published:** 2024-12-30

**Authors:** Ruth Plackett, Ella Ferris

**Affiliations:** 1 Research Department of Primary Care & Population Health, University College London, London, United Kingdom; 2 Department of Epidemiology and Public Health, University College London, London, United Kingdom; PLOS: Public Library of Science, UNITED KINGDOM OF GREAT BRITAIN AND NORTHERN IRELAND

## Abstract

The coronavirus disease 2019 (COVID-19) pandemic subjected the global population to a situation that aroused disproportionate Health Anxiety (HA). However, this association has not been explored in a systematic review or meta-analysis. The aim of this systematic review was to assess the prevalence and determining factors of HA in the general adult population during the COVID-19 pandemic. A systematic search was conducted across the databases MEDLINE, PsychINFO, Embase and Web of Science. Observational studies using the 18-item Short Health Anxiety Inventory to measure HA during the pandemic were included. A narrative synthesis and meta-analysis summarised HA levels in the general adult population, subgroups and by associated factors. Out of 4088 studies, 12 met the inclusion criteria. Meta-analyses revealed a mean HA score of 15.16 (SE = 0.415). Significantly higher HA levels were observed among females, unmarried individuals, and those with pre-existing health conditions. The HA score of 15.16 suggests elevated HA during the COVID-19 pandemic compared to pre-pandemic studies. Understanding which groups may be more affected by HA during pandemics and health crises will enable us to develop more tailored public health strategies to mitigate the psychological effects of future public health crises. Further research is needed to establish causal and longitudinal relationships.

## Introduction

Some degree of health-related concern is deemed acceptable when proportionate to an existing health risk, but health anxiety (HA) can exceed this threshold and reach clinically relevant levels as a mental health disorder [1]. HA, also known as hypochondriasis or illness anxiety, is broadly defined as excessive worry and fear of ill health, irrespective of somatic symptoms [2]. With an estimated prevalence of five percent in the general adult population, HA is relatively common and is housed under the broader category of anxiety disorders [3, 4]. Anxiety disorders are the most prevalent group of mental health conditions and are characterised by disproportionate anxiety, fear and avoidance of perceived threats [5]. In the context of HA, these threats can manifest internally, through bodily sensations, or external factors, including the illness or death of a relative [2, 5]. Public health crises, such as epidemics and pandemics, represent a widescale external threat to the health of populations, serving as a potential risk factor for the development of HA [6].

Clinical features of HA that distinguish it from other types of anxiety disorder and obsessive compulsive disorder (OCD) include: persistent concerns and hypervigilance relating to health, excessive physical self-monitoring, interpretation of physical sensations as evidence of illness, constant reassurance seeking about health, obsessive internet research on health information, and acting as if ill [2]. Until the development of the cognitive-behavioural model of HA by Warwick et al. [7], HA was not recognised as an anxiety-related disorder, and was considered a treatment-resistant condition [7, 8]. Within this cognitive-behavioural model, when an internal or external health trigger is perceived as a threat, anxiety is induced that can lead to bodily hypervigilance, physiological arousal and safety-seeking behaviours [8]. This cycle of anxiety and maladaptive behaviours reinforces the belief of serious illness, sustaining the condition [8]. The psychological impact of HA on individuals can be debilitating in its most severe form, severely impacting daily functioning and quality of life [1]. Meanwhile, the safety-seeking behaviours of HA impose a considerable strain on healthcare services, with an estimated annual cost of £56 million in England alone due to unnecessary healthcare appointments and medical tests [9].

### The COVID-19 pandemic

One of the largest public health crises to date followed the emergence of the highly transmissible coronavirus disease 2019 (COVID-19), which fundamentally transformed healthcare, economies and everyday routines [1]. Formally declared a pandemic on the 11^th^ March 2020 by the World Health Organization (WHO), the COVID-19 virus subjected the global population to an unprecedented, rapidly evolving situation plagued with uncertainty [10, 11]. Combined with a considerable shift in day-to-day life with the introduction of public health measures, such as social isolation, a profound psychological strain was imposed on individuals [12–14]. Although this situation aroused health-related concern in most, these levels were typically proportionate to the threat [15, 16]. However, for some, the response was maladaptive. For example, those with HA were more likely to engage in excessive COVID-19 testing and body temperature checking and seeking frequent reassurance from health professionals that they are not ill [15]. This can lead to unnecessary worry that persists after the threat subsides, leaving individuals with prolonged HA [16]. Existing research has demonstrated an association between pandemic exposure and anxiety levels, with the WHO estimating a 25.6% increase in anxiety disorder cases [17]. Moreover, various demographic, psychological, and social factors have been shown to influence this relationship, with greater anxiety observed among females, younger individuals, lower socioeconomic backgrounds, and those with pre-existing mental and physical health conditions [4, 17–19].

HA in the pandemic influenced health-related behaviours that impacted both individual wellbeing and the collective response to the crisis [20]. Whilst some individuals may have sought frequent medical attention, overcrowding already strained healthcare services, others avoided seeking medical attention due to transmission concerns within healthcare facilities [21]. Consequently, in some cases essential care was delayed, increasing the likelihood of more severe and prolonged health issues, which have been associated with an increase in avoidable patient deaths [21]. Further, the overlap between symptoms of COVID-19, such as cough, fever, and headache, and those of the common cold and influenza created diagnostic challenges [22].

### Research gaps

Despite the increasing relevance of HA throughout the COVID-19 pandemic, this relationship has been less extensively explored relative to other anxiety outcomes. Whilst individual studies have investigated the prevalence and determinants of HA during the pandemic, there is currently no systematic review to summarise the findings. Notably, studies on past epidemics and pandemics, such as the 2014 Ebola outbreak, have consistently observed an increase in HA among populations during public health crises [23–25]. Given the widespread likelihood of HA during the COVID-19 pandemic, a systematic review is needed to consolidate existing knowledge and identify key trends. Prior to the COVID-19 pandemic, a limited number of systematic reviews investigated HA but none have examined how COVID-19 impacted HA [26–28]. Among these studies, factors such as age, gender and comorbidity were discussed as having a potential predictive role in HA. Understanding the prevalence and determinants of HA during public health crises will help to inform mental and public health strategies that are tailored to the needs of individuals and wider society.

### Aims

The aims of this systematic review and meta-analysis were to assess the prevalence and determining factors of HA in the general adult population during the COVID-19 pandemic.

## Methods

This systematic review and meta-analysis has been guided by the Preferred Reporting Items for Systematic Reviews and Meta-Analyses (PRISMA) 2020 checklist (S1 Checklist) and is registered on PROSPERO (CRD42023450777) [29].

### Search strategy

A comprehensive literature search was conducted across the electronic databases MEDLINE, PsychINFO, Embase and Web of Science Core Collection in June 2023. The search strategy (S1 Text) included medical subject headings and synonyms for the core concepts of "health anxiety" and "COVID-19". Results were filtered to include articles published from 2020 onwards, following the declaration of COVID-19 as a pandemic on the 11^th^ March 2020 by the WHO [11].

### Inclusion and exclusion criteria

The inclusion and exclusion criteria are shown in Table 1. Any measurement of HA was included in the search strategy but during screening it was decided to only include studies measuring HA using the Short Health Anxiety Inventory-18 (SHAI-18) and to exclude studies employing non-specific or other HA psychometric tools. This was because many of these other tools were author developed measures that could not demonstrate sufficient reliability and validity. The SHAI-18 was identified as the most appropriate psychometric tool to answer the research question as it assesses both healthy and physically ill adults [26] it has been adapted for different cultural settings and was most commonly used among studies, enabling more accurate comparison across studies [30, 31]. Furthermore, the SHAI-18 is a valid psychometric tool with good to excellent internal reliability across samples (α = 0.74–0.96), distinct from alternative psychometric tools, such as the HAQ, which have unclear validity and reliability [26]. Only observational studies were included as we sought to understand the prevalence and determinants of HA. Higher scores on the SHAI-18 equate to higher levels of HA.

**Table 1:** Inclusion and exclusion criteria.

	Inclusion criteria	Exclusion criteria
**Publication Date**	2020-present	Before 2020
**Population**	General adult population (≥18 years)	Individuals under 18 years Subgroups (such as students)
**Exposure**	COVID-19 pandemic	Not COVID-19 pandemic
**Outcome**	Health-specific anxiety	Mental health states not primarily related to health (including general COVID-19 anxiety)
**Outcome Measure**	HA measured by the Short Health Anxiety Inventory-18 (SHAI-18)	Psychometric tools that do not explicitly measure HA Individual SHAI-18 items
**Study Type**	Observational studies	Interventional studies Qualitative studies Psychometric tool validation studies
**Publication Type**	Peer-reviewed articles Full-text available	Case studies Commentaries Editorial letters Theses Grey literature Reviews Meta-analyses Articles not published in English

### Screening

Results produced from the final search strategy were exported to the reference management software EndNote, where duplicate records were removed. Remaining records were exported to Excel, where title and abstract screening were conducted using the inclusion and exclusion criteria (Table 1). Title and abstract screening were undertaken by a second reviewer (RP) for 10% of records selected using random number assignment in Excel. Reviewer disagreements were resolved through discussion. The level of agreement was good (Cohen’s kappa score of 0.77). Remaining records then underwent full-text screening, with 10% assessed by a second reviewer.

### Data extraction

Data collection was carried out using an excel-based data extraction form adapted from guidance by the Centre for Reviews and Dissemination [32]. A second reviewer (RP) checked 10% of the data extraction records to ensure rigor and consistency. Relevant information was recorded for the study characteristics (year, author, country of origin, publication type, sample characteristics, aims, methods, HA outcomes, factors affecting HA and study limitations). HA prevalence data was extracted, where available, in the form of mean or median total SHAI-18 scores for each study population, with subgroup scores extracted when provided. Data comparing baseline and comparator HA scores within cohort studies could not be retrieved as this measurement was absent in all studies. The measures of association and associated p-values were extracted for the relationship between HA and various factors, which were categorised under ‘demographic’, ‘psychological’, ‘policy’, ‘relational’, ‘behavioural’ and ‘social’ based on the data provided. Effect sizes (Cohen’s d) were calculated where sufficient data was available [33].

### Meta-analysis

The software package MedCalc v22.009 was employed to quantitatively analyse total and subgroup mean SHAI-18 scores in a random-effects meta-analysis, where sufficient data was provided [34]. A random-effects model (DerSimonian and Laird) was used to account for the assumption that the studies are estimating distinct yet related effects [35]. Due to the small number of cohort studies [36–39] and heterogeneity in their data collection periods, a meta-analysis of SHAI-18 scores over time was not conducted. Separate random-effects models were carried out for subgroups for which multiple studies reported mean scores: gender, marital status, pre-existing physical and mental health conditions. Heterogeneity was assessed using I² statistics. A significance level of α = 0.05 was applied, and 95% confidence intervals were reported for effect sizes.

### Narrative synthesis

A narrative synthesis was conducted to explore in individual studies the associations between other factors and HA. These were categorised into six factors: demographic factors such as age and location; psychological factors relating to thoughts and emotions such as feelings of hopelessness and uncertainty; social factors relating to an individual’s wider social network such as community cohesion; relational factors referring to an individual’s relationships with others such as their parental status and having a vulnerable relative; behavioural factors referring to behaviours such as news consumption and policy factors related policies that were put in place during the pandemic, such as stay at home orders and lockdowns.

### Quality assessment

The Crowe Critical Appraisal Tool (CCAT) v1.4 was employed to evaluate the methodological quality of the included studies as it allowed for the appraisal of cross-sectional study designs [40]. All studies were assessed across eight categories: preliminaries, introduction, design, sampling, data collection, ethical matters, results and discussion. Each category was scored 0 to 5, with studies rated low (≤20), medium (<30) or high (≤40)quality based on a maximum total score of 40 in accordance with published guidance [41].

## Results

### Study results

**Fig 1:**
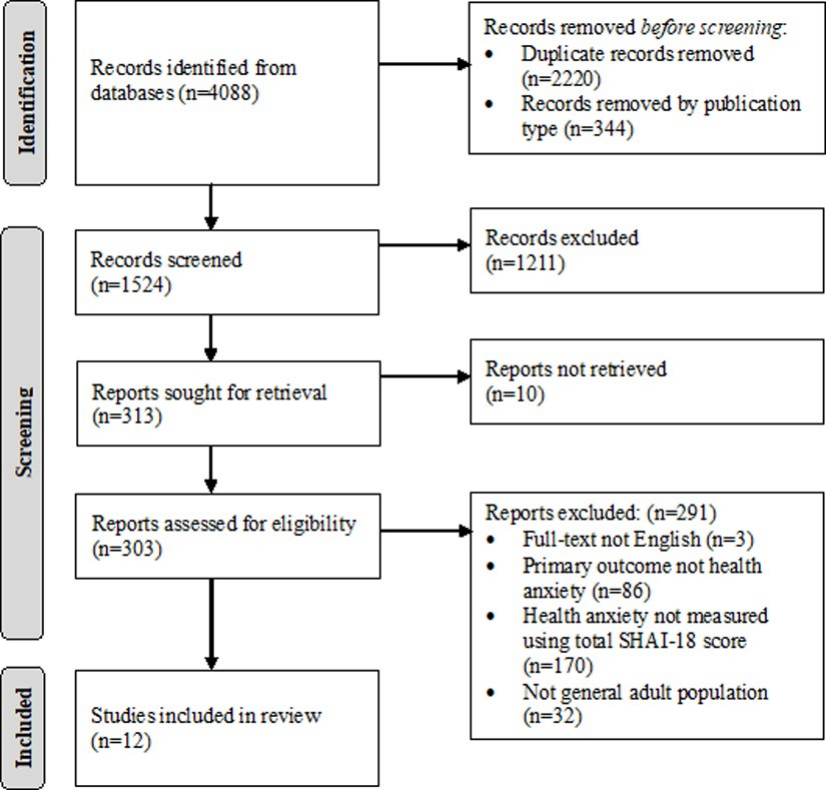
PRISMA flow diagram for search results.

The search strategy identified 4088 results (see S1 Table for a list of all the studies identified). After removing duplicates and articles based on publication type, title and abstract screening was conducted on the remaining 1524 results. A high number of articles underwent full-text screening (n = 303) due to unclear HA measurement at abstract-level. Full-text screening excluded a further 291 studies, producing a final selection of 12 studies (Fig 1). The main reason for exclusion was employing an alternative psychometric tool to the SHAI-18.

### Study characteristics

Table 2 provides a summary of the key characteristics of the included studies. Studies were published between 2020 and 2023, with most conducted in Turkey (n = 5, 42%), the United States (n = 3, 25%) and the United Kingdom (n = 2, 17%). The predominant study design was cross-sectional (n = 8, 67%), with the remaining studies employing a cohort design (n = 4, 33%) in which HA levels were compared to a baseline level either before or during the COVID-19 pandemic [36–39]. Most studies reported a mean total SHAI-18 score, with the exception of Canli et al. [42] reporting mean total scores by age group and Kirmizi et al. [43] reporting median total scores by gender. Few studies (n = 4, 33%) reported an SHAI-18 clinical cut-off score, with each study utilising a different score [36, 38, 44, 45]. Data collection occurred exclusively in 2020 for most studies (n = 10, 83%), with all but Yalcin et al. [46] specifying a data collection period; however, it is assumed that sampling occurred during the same period. The most examined factors for a relationship with HA were age, gender, marital status and pre-existing health conditions.

**Table 2:** Characteristics of included studies by study design.

Study	Country	Study Design	Sample Size	Age		Gender [%]			Ethnicity [% white ethnicity]	Data Collection Period	Mean Total SHAI±SD	Cut-off		Determinants Examined
				Range	Mean±SD or Median (Q1-Q3) or %	Male	Female	Other				Cut-off	n ≥ (%)	
Bredemeier et al. [36]	United States	Cohort	301	18–65	30.90^a^*	57.10	42.90	N/A	N/S	06/20	15.51±7.96^a^	27.00	30 (10.00)	Anxiety sensitivity, Intolerance of uncertainty
										12/20	15.72±8.37^a^		33 (11.00)	
Chan et al. [37]	Hong Kong	Cohort	279	18–64	27.35±9.40^a^	26.20	73.80	N/A	N/S	03/07/19-22/07/19 **	15.33±6.31^a^	N/A		Age, Gender, Negative COVID-19 interpretation, Negative illness interpretation, Pre-pandemic HA, Pre-pandemic illness interpretation
										03/07/20-24/07/20	15.52±6.70^a^			
Heinen et al. [38]	United Kingdom	Cohort	199	18–82	43.20±16.50^a^	26.90	73.10	N/A	82.70	09/07/20-15/05/21	12.90±7.50^a^	18.00	N/S	Gender, Pre-existing physical and mental health condition
										3 months from first collection	12.90±8.30^a^			
Tull et al. [39]	United States	Cohort	364	20–74	41.25±12.02^a^	47.50	51.40	1.10	84.90	27/03/20-05/04/20	N/A	N/A		N/A^†^
										27/04/20-21/05/20	13.39±8.92^a^			
Canli et al. [42]	Turkey	Cross-sectional	874	18–30	59.40^c^	26.50	73.50	N/A	N/S	04/20-05/20	Subgroup scores only	N/A		Age, Education, Gender, Marital status, Occupation, Pre-existing physical or mental health conditions
				31–40	20.70^c^									
				41–50	14.20^c^									
				>51	5.70^c^									
	
Kirmizi et al. [43]	Turkey	Cross-sectional	170	18–60	Male 29.00 (24–29)^b^	50.00	50.00	N/A	N/S	01/06/20-10/06/20	Subgroup scores only	N/A		Gender
					Female 28.00 (23–28)^b^									
Kizilkurt et al. [47]	Turkey	Cross-sectional	1046	18–65	37.10±12.80^a^	66.00	34.00	N/A	N/S	28/03/20-04/04/20	17.10±6.90^a^	N/A		Gender, Marital status, Parental Status, Pre-existing physical or mental health condition, Location, Hopelessness, Self-confidence, COVID-19 news viewing, Precautionary behaviour, Social media use, COVID-vulnerable relative
Ozdin et al. [48]	Turkey	Cross-sectional	343	18–50	37.16±10.31^a^	50.70	49.20	N/A	N/S	14/04/20-16/04/20	15.10±7.00^a^	N/A		Age, Gender, Marital status, Pre-existing physical or mental health condition, Location, Friends or relatives with COVID-19, Living with individual aged 60+, Pandemic work
Svensson et al. [44]	United Kingdom	Cross-sectional	2329	18–87	48.08±13.39^a^	17.60	82.40	N/A	N/S	03/20-05/20	14.08±7.32^a^	14.48	943 (40.50)	Community cohesion, Days under lockdown
Tull et al. [49]	United States	Cross-sectional	500	20–74	40.00±11.6^a^	51.80	47.00	1.20	85.00	27/03/20–05/04/20	32.29±9.32^a^***	N/A		Stay-at-home order, Perceived COVID-19 impact
Wechsler et al. [45]	Germany	Cross-sectional	396	18–30	34.80^c^	29.30	70.20	0.50	N/S	10/04/20-27/04/20	17.19±11.07^a^	23.93	113 (28.50)	Age, Gender
				31–40	25.50^c^									
				41–50	13.60^c^									
				51–65	19.90^c^									
				>66	6.10^c^									
Yalcin et al. [46]	Turkey	Cross-sectional	8276	18–65	39.86±13.13^a^	52.70	47.30	N/A	N/S	N/S	15.31±9.94^a^	N/A		Fear of COVID-19, Perceived disease vulnerability

N/S: Not specified. N/A: Not applicable.

*SD not specified.

**Data collection from pre-COVID-19 study.

***SHAI scored 1–4.

^†^Only assessed for SHAI categories.

^a^Mean±SD.

^b^Median (Q1-Q3).

^c^Percentage

### Sample characteristics

Sample characteristics are summarised in Table 2. Across all included studies, sample sizes ranged from 170 to 8276 and age from 18 to 87. Almost half of studies had predominantly female samples (n = 5, 42%), comprising between 70 and 82% of the study population. Ethnicity was only reported by three studies (25%), comprising a white ethnicity majority of over 80% in each study.

### Quality assessment

The results from the quality assessment of studies using the CCAT form are displayed in S2 Table. Scores ranged from 27 to 35, with a mean score of 32. Most studies (n = 10, 83%) were high quality. The remaining two studies [37, 42] were rated moderate quality due to issues with sampling.

### Meta-analysis

A meta-analysis was conducted on the nine studies that provided a mean total SHAI-18 score. Three studies [42, 43, 49] were excluded as two [42, 43] provided total scores by subgroup and Tull et al. [49] scored SHAI-18 items from 1 to 4 and did not provide enough data to standardise to 0 to 3 scoring. Additional meta-analyses were conducted for subgroups where mean total scores were provided by multiple studies (Table 3). Age was excluded from meta-analysis due to minimal data and heterogeneity in reported age categories. Similarly, a meta-analysis of HA by SHAI-18 clinical cut-off scores was not conducted due to the limited and heterogeneous cut-off scores reported.

**Table 3:** Health anxiety by subgroups and associated factors.

Factor type	Factor	Study	Subgroup	Sample size n (%)	Mean Total SHAI±SD or Median (Q1-Q3)	Measure of Association	P-value	Effect size (d)	Interpretation
Demographic	Age	Canli et al. [42]	18–30 years	519 (59.38)	16.53±6.65	16.25^a^	<0.001**	0.2753	Younger age had significantly greater HA, with the highest levels among those aged 18–30
			31–40 years	181 (20.71)	14.75±7.18				
			41–50 years	124 (14.19)	14.89±7.49				
			>51 years	50 (5.72)	14.26±7.28				
		Chan et al. [37]	None	N/A	N/A	-0.122 (0.035)^c^	0.001*	N/A	Younger age had significantly greater HA
		Ozdin et al. [48]	18–49	N/S	15.10±6.80	N/S	0.458	N/A	Non-significant association between age and HA
			≥50	N/S	14.70±8.00				
		Wechsler et al. [45]	None	N/A	N/A	3.46^a^	0.503	0.1878	Non-significant association between age and HA
	Education	Canli et al. [42]	Elementary-secondary	25 (2.86)	19.88±8.83	8.62^a^	0.070	0.1996	Non-significant association between education level and HA
			High school	101 (11.56)	15.91±8.70				
			Associate’s degree	58 (6.64)	16.46±7.17				
			Bachelor’s degree	597 (68.31)	15.73±6.61				
			Graduate degree	93 (10.64)	14.61±6.08				
	Gender	Canli et al. [42]	Male	232 (26.54)	13.66±6.56	56174.50^b^	<0.001**	0.035	Females had significantly greater HA
			Female	642 (72.46)	16.57±6.95				
		Chan et al. [37]	None	N/A	N/A	−0.865 (0.746)^c^	0.247	N/A	Non-significant association between gender and HA
		Heinen et al. [38]	Male	87 (26.90)	10.50±6.40	0.180^d^	0.001*	N/A	Females had significantly greater HA
			Female	237 (73.10)	13.80±7.60				
		Kirmizi et al. [43]	Male	85 (50.00)	16 (13–21)	N/S	<0.001**	N/A	Females had significantly greater HA
			Female	85 (50.00)	13 (9–15)				
		Kizilkurt et al. [47]	Male	690 (66.00)	16.10±6.70	N/S	<0.001**	N/A	Females had significantly greater HA
			Female	356 (34.00)	17.60±6.90				
		Ozdin et al. [48]	Male	174 (50.70)	14.20±6.20	0.105^d^	0.030*	N/A	Females had significantly more HA
			Female	169 (49.20)	15.90±7.60				
		Wechsler et al. [45]	None	N/A	N/A	1.72^a^	0.486	0.132	Non-significant association between gender and HA
	Marital status	Canli et al. [42]	Unmarried	544 (62.24)	16.15±6.71	82339.50^b^	0.040*	0.139	Unmarried individuals had significantly greater HA
			Married	330 (37.76)	15.23±7.35				
		Kizilkurt et al. [47]	Unmarried	471 (45.00)	17.90±6.90	N/S	<0.001**	N/A	Unmarried individuals had significantly more HA
			Married	575 (55.00)	16.40±6.90				
		Ozdin et al. [48]	Unmarried	125 (36.40)	16.10±7.80	N/S	0.144	N/A	Non-significant association between marital status and HA
			Married	218 (63.50)	14.50±6.40				
	Occupation	Canli et al. [42]	Student	331 (37.87)	16.32±5.97	17.18^a^	0.040*	0.2832	Significant association between occupation and HA, with the highest levels among the unemployed or retired
			Healthcare worker	187 (21.40)	16.01±7.08				
			Educator	130 (14.87)	14.75±6.78				
			Private sector	111 (12.70)	14.73±7.63				
			Public worker	36 (4.12)	13.44±6.96				
			Unemployed-retired	79 (9.04)	17.44±9.13				
		Svensson et al. [44]	Managers, directors and senior official	126 (5.41)	12.23±6.01	None	N/A	N/A	N/A
			Professional occupations	762 (32.72)	14.16±7.21				
			Associate professional and technical occupations	215 (9.23)	12.92±6.18				
			Administrative and secretarial occupation	224 (9.62)	14.62±7.24				
			Skilled trades occupations	73 (3.13)	13.71±6.40				
			Caring, leisure and other service occupations	175 (7.51)	15.08±7.74				
			Sales and customer service occupations	74 (3.18)	14.00±8.05				
			Process, plant and machine operatives	17 (0.73)	13.35±7.95				
			Low skilled elementary occupations	75 (3.22)	14.47±9.00				
			Students	81 (3.48)	17.08±6.91				
			Retired	369 (15.84)	12.24±6.45				
			Not working	56 (2.40)	17.96±9.20				
			Home duties	83 (3.56)	18.50±8.62				
	Pre-existing physical health condition	Canli et al. [42]	Yes	131 (15.00)	17.35±7.99	42793.00^b^	0.020*	0.353	Individuals with pre-existing physical health conditions had significantly greater HA
			No	743 (85.00)	15.53±6.74				
		Heinen et al. [38]	Yes	87 (26.90)	10.50±6.40	0.210^d^	<0.001**	N/A	Individuals with pre-existing physical health conditions had significantly greater HA
			No	237 (73.1)	13.80±7.60				
		Kizilkurt et al. [47]	Yes	178 (17.00)	18.10±7.10	N/S	0.030*	N/A	Individuals with pre-existing physical health conditions had significantly greater HA
			No	868 (83.00)	16.80±6.90				
		Ozdin et al. [48]	Yes	54 (15.70)	17.90±7.60	0.160^d^	0.001*	N/A	Individuals with pre-existing physical health conditions had significantly greater HA
			No	289 (84.30)	14.60±6.70				
	Location	Kizilkurt et al. [47]	Marmara	743 (71.00)	17.30±7.10	N/S	0.290	N/A	Non-significant association between location and HA
			Aegean	63 (6.00)	16.40±5.30				
			Mediterranean	31 (3.00)	15.90±5.80				
			Black Sea	21 (2.00)	18.30±8.60				
			Central Anatolia	84 (8.00)	16.30±6.20				
			East Anatolia	31 (3.00)	18.10±6.10				
			Southeast Anatolia	42 (4.00)	15.30±7.50				
		Ozdin et al. [48]	Urban	278 (81.00)	15.20±7.10	N/S	0.550	N/A	Non-significant association between location and HA
			Rural	65 (18.90)	14.40±6.30				
		Svensson et al. [44]	Northwest	872 (37.44)	14.73±8.01	None	N/A	N/A	N/A
			Southeast	275 (11.81)	12.97±6.33				
			Southwest	208 (8.93)	13.16±6.57				
			Greater London	195 (8.37)	14.61±7.24				
			Scotland	149 (6.40)	13.70±7.47				
			East of England	122 (5.24)	13.43±6.71				
			West Midlands	117 (5.02)	13.85±7.24				
			Yorkshire	114 (4.89)	13.71±6.74				
			East Midlands	101 (4.34)	13.43±6.42				
			Wales	92 (3.95)	13.86±6.31				
			Northeast	64 (2.75)	16.64±7.91				
			Northern Ireland	20 (0.86)	12.10±5.41				
Psychological	Anxiety sensitivity	Bredemeier et al. [36]	None	N/A	N/A	0.070 ^d^	>0.05	N/A	Non-significant association between anxiety sensitivity and HA
	Fear of COVID-19	Yalcin et al. [46]	None	N/A	N/A	0.359 (0.008)^c^	<0.001**	N/A	Greater fear of COVID-19 significantly associated with greater HA
	Hopelessness	Kizilkurt et al. [47]	None	N/A	N/A	0.140^d^	<0.001**	N/A	Greater hopelessness significantly associated with greater HA
	Intolerance of uncertainty	Bredemeier et al. [36]	None	N/A	N/A	0.130^d^	<0.01*	N/A	Greater intolerance of uncertainty significantly associated with greater HA
	Negative COVID-19 interpretations	Chan et al. [37]	None	N/A	N/A	0.071 (-0.024)^c^	0.003*	N/A	Negative COVID-19 interpretation significantly associated with greater HA
	Negative illness interpretations	Chan et al. [37]	None	N/A	N/A	0.082 (0.026)^c^	0.002*	N/A	Negative illness interpretation significantly associated with greater HA
	Perceived COVID-19 impact	Tull et al. [49]	None	N/A	N/A	1.600 (0.400)^c^	0.010*	N/A	Negative COVID-19 impact perception significantly associated with greater HA
	Perceived disease vulnerability	Yalcin et al. [46]	None	N/A	N/A	0.098 (0.009)^c^	<0.001**	N/A	Negative disease vulnerability perception significantly associated with greater HA
	Self-confidence	Kizilkurt et al. [47]	None	N/A	N/A	-0.310^d^	<0.001**	N/A	Lower self-confidence significantly associated with greater HA
	Pre-existing Mental health condition	Canli et al. [42]	Yes	33 (3.78)	21.45±8.72	8551.00^b^	<0.001**	0.255	Individuals with pre-existing mental health conditions had significantly greater HA
			No	841 (96.22)	15.58±6.80				
		Heinen et al. [38]	Yes	46 (23.00)	15.10±8.80	0.300^d^	<0.001**	N/A	Individuals with pre-existing mental health conditions had significantly greater HA
			No	153 (77.00)	12.10±6.70				
		Kizilkurt et al. [47]	Yes	261 (25.00)	19.50±7.40	N/S	<0.001**	N/A	Individuals with pre-existing mental health conditions had significantly greater HA
			No	785 (75.00)	16.30±6.60				
		Ozdin et al. [48]	Yes	75 (21.80)	18.00±8.20	0.176^d^	0.001*	N/A	Individuals with pre-existing mental health conditions had significantly greater HA
			No	268 (78.20)	14.30±6.40				
	Pre-pandemic HA	Chan et al. [37]	None	N/A	N/A	0.472 (0.055)^c^	<0.001**	N/A	Greater pre-pandemic HA significantly associated with greater HA
	Pre-pandemic illness interpretation	Chan et al. [37]	None	N/A	N/A	−0.020 (0.024)^c^	0.400	N/A	Non-significant association between pre-pandemic illness interpretation and HA
Social	Community cohesion	Svensson et al. [44]	None	N/A	N/A	-0.180 (-0.220, -0.130)^c^	<0.001**	N/A	Lower community cohesion significantly associated with greater HA
Behavioural	COVID-19 news viewing	Kizilkurt et al. [47]	None	N/A	N/A	0.110^d^	0.002*	N/A	Greater COVID-19 news viewing significantly associated with greater HA
	Precautionary behaviours	Kizilkurt et al. [47]	Yes	638 (61.00)	18.10±6.90	N/S	<0.001**	N/A	Greater precautionary behaviours significantly associated with greater HA
	Social media use	Kizilkurt et al. [47]	None	N/A	N/A	0.10^d^	0.002*	N/A	Greater social media use significantly associated with greater HA
	Going outside	Ozdin et al. [48]	Yes	282 (82.20)	15.00±7.00	N/S	0.342	N/A	Non-significant association between going outside and HA
			No	61 (17.70	15.50±6.80				
	Working after pandemic	Ozdin et al. [48]	Yes	161 (64.40)	14.80±6.70	N/S	0.542	N/A	Non-significant association between post-pandemic work and HA
Policy	Days under lockdown	Svensson et al. [44]	None	N/A	N/A	-0.040 (-0.050, -0.020)^c^	<0.001**	N/A	More days under lockdown significantly associated with lower HA
	Stay-at-home order	Tull et al. [49]	None	N/A	N/A	2.78 (1.08)^c^	0.010*	N/A	Stay-at-home order significantly associated with greater HA
Relational	Friends or relatives with COVID-19	Ozdin et al. [48]	None	N/A	N/A	0.064^d^	0.224	N/A	Individuals with friends or relatives with COVID-19 had significantly greater HA
	Parental status	Kizilkurt et al. [47]	Have children	502(48.00)	17.80±6.90	N/S	0.003*	N/A	Parents had significantly greater HA
			No children	544 (52.00)	16.50±6.80				
	Living with individual aged 60+	Ozdin et al. [48]	Yes	72 (20.90)	14.60±7.70	N/S	0.403	N/A	Non-significant association between individuals living with someone aged 60+ and HA
			No	271 (79.00)	15.20±6.80				
	Vulnerable relative to COVID-19	Kizilkurt et al. [47]	Yes No	732 (70.00) 314 (30.00)	17.40±6.70 15.40±7.30	N/S	0.040*	N/A	Individuals with a vulnerable relative had significantly greater HA

HA: Health anxiety. N/S: Not specified. N/A: Not applicable.

*p<0.05.

**p<0.001.

^a^Chi-squared statistic.

^b^Mann-Whitney U statistic.

^c^Unstandardised beta coefficient.

^d^Standardised beta coefficient

#### Mean total health anxiety scores

The nine studies [36–39, 44–48] were combined in a random effects model meta-analysis. The results revealed a pooled SHAI-18 mean score of 15.16 (SE = 0.415, 95% CI = 14.343–15.970). Significant heterogeneity was present between studies (I^2^ = 95.63%, p<0.001).

#### Health anxiety scores by gender

**Fig 2:**
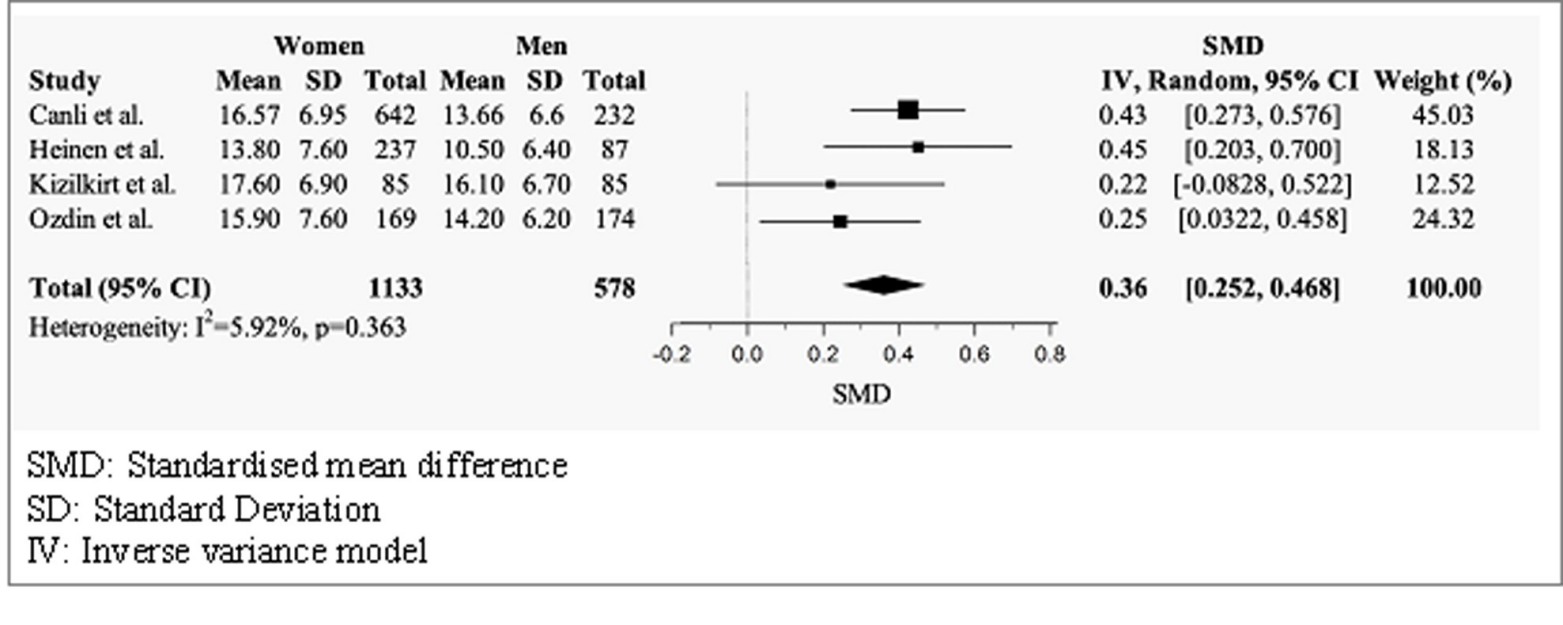
Forest plot of mean total SHAI-18 scores by gender.

Four studies compared male and female mean total SHAI-18 scores (Fig 2) [38, 42, 47, 48]. The random effects model indicated that females had significantly higher scores than males, with a positive small effect size (g = 0.36, 95% CI = 0.252–0.468). There was no significant heterogeneity across studies (I^2^ = 5.92%, p = 0.363).

#### Health anxiety scores by marital status

**Fig 3:**
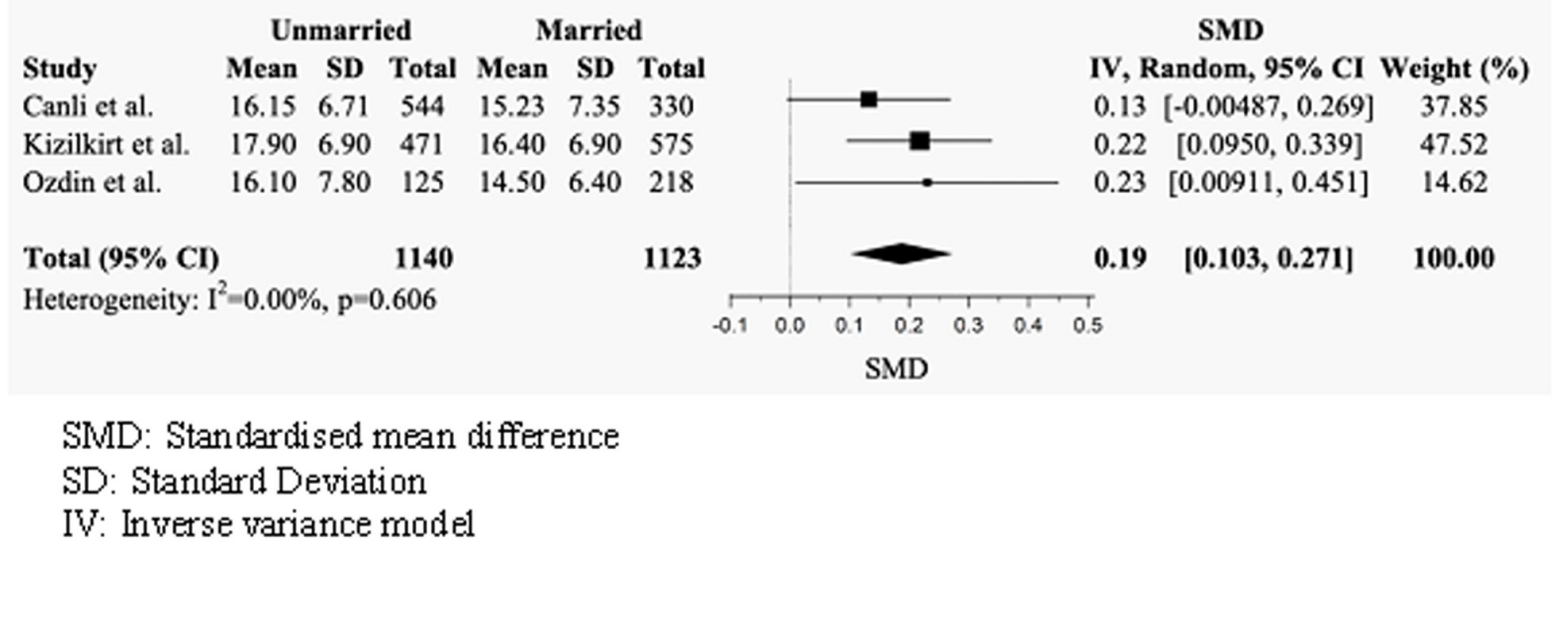
Forest plot of mean total SHAI-18 scores by marital status.

Three studies compared the mean total SHAI-18 scores of married and unmarried individuals (Fig 3) [42, 47, 48]. The random effects model indicated that unmarried individuals had significantly higher scores than married individuals, with a positive small effect size (g = 0.19, 95% CI = 0.103–0.271). No significant heterogeneity was observed across studies (I^2^ = 0.00%, p = 0.606).

#### Health anxiety scores by pre-existing physical health status

**Fig 4:**
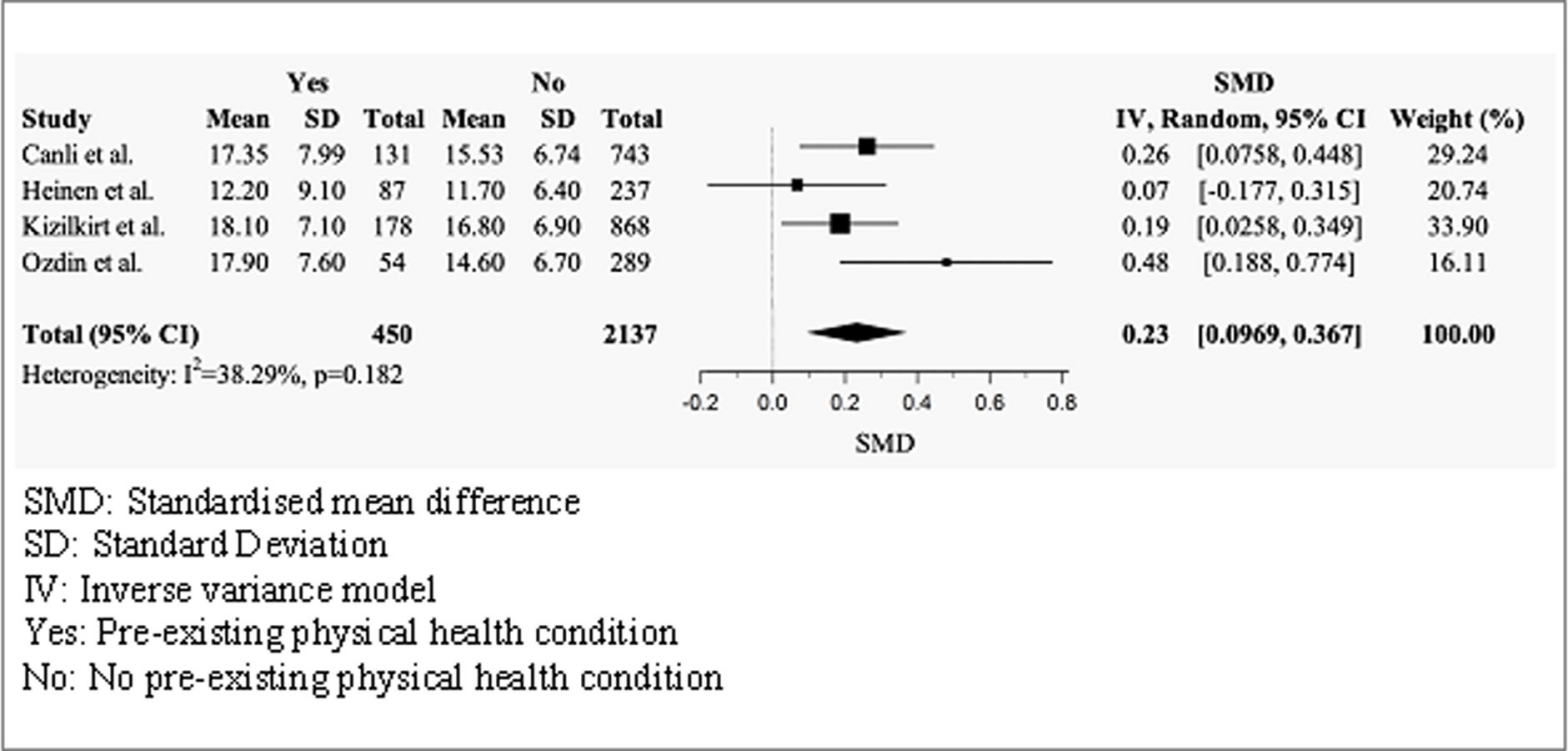
Forest plot of total mean SHAI-18 scores by physical health status.

Four studies compared the mean total SHAI-18 scores of individuals with and without a pre-existing physical health condition (Fig 4) [38, 42, 47, 48]. The random effects model produced a positive small effect size (g = 0.23, 95% CI = 0.0969–0.367), indicating that individuals with a pre-existing physical health condition had significantly higher scores than individuals without. No significant heterogeneity was observed across studies (I^2^ = 38.29%, p = 0.182).

#### Health anxiety scores by pre-existing mental health status

**Fig 5:**
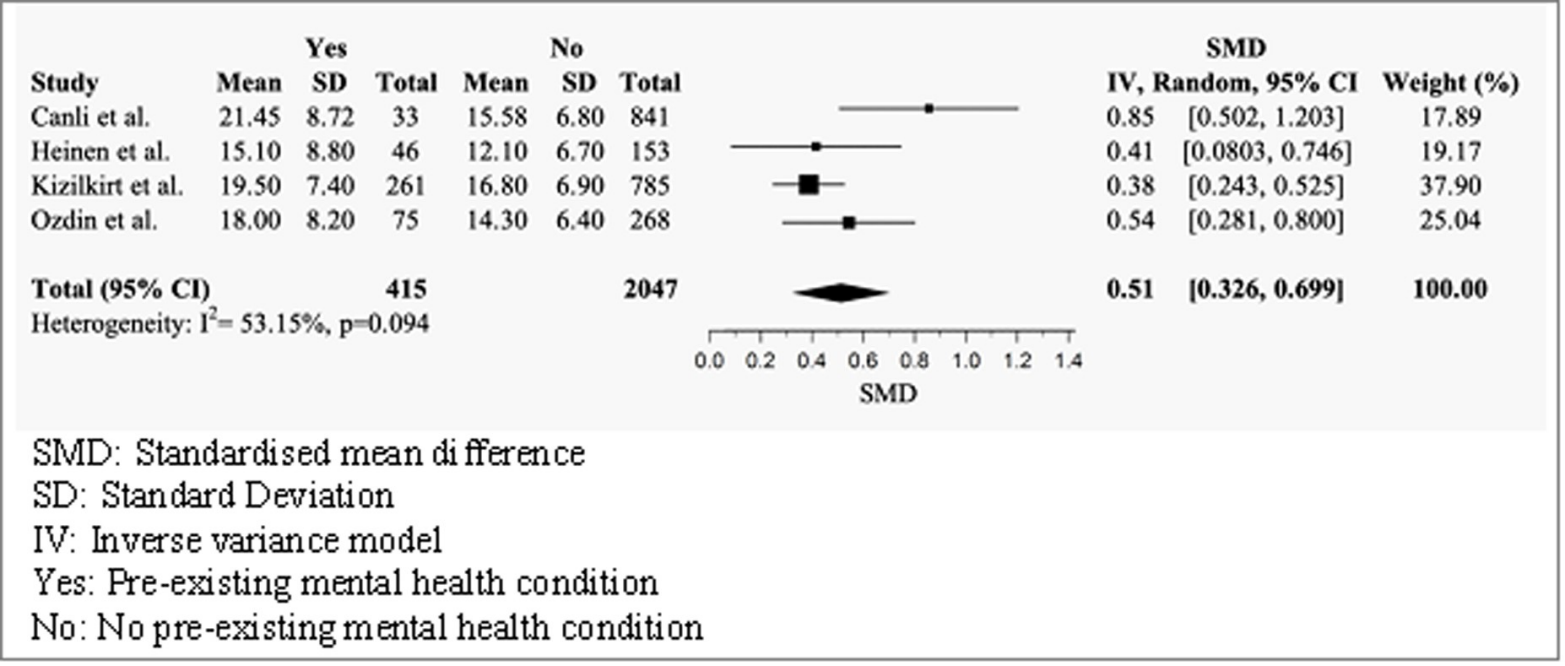
Forest plot of total mean SHAI-18 scores by mental health status.

Four studies compared the mean total SHAI-18 scores of individuals with and without a pre-existing mental health condition (Fig 5) [38, 42, 47, 48]. The random effects model produced a positive medium effect size (g = 0.51, 95% CI = 0.326–0.699), indicating that individuals with a pre-existing mental health condition had significantly higher scores than individuals without. No significant heterogeneity was observed across studies (I^2^ = 53.15%, p = 0.094).

### Narrative synthesis

#### HA over time

Three cohort studies reported mean SHAI-18 scores over time [40–42]. Two studies found very small increases in mean anxiety scores over time, with one study examining a one-year period and the other a six-month period [40, 41]. One study found no mean difference in mean anxiety scores over a 3-month period [42].

#### Associations with demographic factors

The four studies [37, 42, 45, 48] investigating age and HA observed mixed findings, with two studies [37, 42] reporting younger age to be significantly associated with greater HA but effect sizes were small [42, 45]. Two studies [42, 44] explored the association between occupation and HA. Only Canli et al. [42] provided sufficient data to quantify the relationship and revealed a significant association, with the highest HA levels among the unemployed or retired. Location was investigated as an associated factor by three studies [44, 47, 48]. Svensson et al. [44] reported insufficient data, with the other two studies [47, 48] revealing a non-significant association. Education was only investigated by one study that found a non-significant relationship between education level and HA [42].

#### Associations with psychological factors

Significant associations with HA were found among individual studies for fear of COVID-19 [46], hopelessness [47], intolerance of uncertainty [36], negative COVID-19 interpretation [37], negative illness interpretation [37], perceived COVID-19 impact [49], perceived disease vulnerability [46], self-confidence [47] and pre-pandemic HA [37]. However, non-significant associations were found for anxiety sensitivity [36] and pre-pandemic illness interpretation [37].

#### Associations with social factors

One study found a significant association between HA and poor community cohesion [44].

#### Associations with relational factors

Within individual studies, significant associations with HA were found for having a vulnerable relative, and parents exhibited significantly greater HA than non-parents [47]. However, non-significant associations with HA were found for having friends or relatives with COVID-19 and living with an individual aged 60 or over [48].

#### Associations with behavioural factors

One study found, significant associations between HA and greater consumption of COVID-19 news, greater social media use and exhibiting more precautionary behaviours [47]. However, another study found non-significant associations between going outside and working after the pandemic and HA [48].

#### Associations with policy factors

Experiencing HA was associated with a greater number of days under lockdown [44] and stay-at-home orders [49].

## Discussion

This systematic review and meta-analysis is the first to comprehensively assess the prevalence and determinants of HA in the general adult population during the COVID-19 pandemic. Meta-analysis revealed an overall mean SHAI-18 score of 15.16. Being female, unmarried or having pre-existing physical and mental health conditions was associated with greater HA during the pandemic. Individual studies observed generally significant effects on HA for psychological factors. However, other factors showed more mixed results, with factors such as poor community cohesion and COVID-19 news viewing demonstrating a more significant effect on HA than other factors, including having friends or relatives with COVID-19.

### Prevalence of health anxiety

The mean score of 15.16 is higher than those reported for non-clinical samples in literature conducted before the COVID-19 pandemic. In their systematic review and meta-analysis, Alberts et al. [26] found a mean of 12.41 (SD = 6.81) across 10 studies. Furthermore, Salkovskis et al. [50] found a similar mean of 12.20 (SD = 6.20) when validating the SHAI-18 in a non-clinical sample. Our results show a mean score almost 3 units above those previously reported in non-clinical samples, suggesting elevated HA during the COVID-19 pandemic [26]. This finding is consistent with recent studies, which have reported heightened levels of anxiety, depression and psychological distress in the general adult population compared to before the pandemic [12, 51, 52]. The absence of a standardised clinically significant cut-off score for the SHAI-18, unlike more widely recognised psychometric tools to assess mental health outcomes, has led to inconsistent score interpretation across studies [26]. The original authors of the SHAI-18 provided no clinical cut-off and existing literature has employed various cut-off scores, with some studies setting thresholds as low as 15 to distinguish between typical levels of health-related concern and significant HA [53, 54]. Few studies included in this review reported a cut-off score, with each reporting a different score. This heterogeneity in cut-off scores precluded the establishment of an overall prevalence of clinically significant HA through meta-analysis, highlighting the need for standardised scoring in future research to facilitate more consistent and comparable assessments of HA. However, it should be noted that existing research describes HA as a continuum that should be described dimensionally, negating dichotomous categorisation [55].

### Factors associated with health anxiety

The observed gender-specific differences in HA that we found strongly aligns with the emerging body of pandemic research, which consistently reports heightened anxiety levels, particularly among females [17–19, 56, 57]. Conversely, pre-pandemic studies have demonstrated inconsistent findings regarding an association between gender and health-specific anxiety [4, 27, 58, 59]. Possible explanations for the higher levels of HA observed in this review are explored by several included studies. Heinen et al. [38] acknowledges that females are reported to experience heightened sensitivity to bodily sensation, which increases their perception and reaction to internal threats. This sensitivity is assessed under item 3 of the SHAI-18; however, an itemised breakdown of scores was absent in all twelve studies. Therefore, future research may wish to explore itemised HA scores to explore the differential presentation of HA by gender, particularly during public health crises.

Although this review observed higher levels of HA in unmarried individuals, literature exploring this relationship is limited. However, broader research in the field has demonstrated an indirect relationship between marital status and mental health outcomes through loneliness, whereby unmarried individuals experience greater loneliness, leading to poorer mental health outcomes [60–62]. In the context of the pandemic, loneliness is emerging as a crucial determining factor for mental health outcomes, suggesting that further research should explore this pathway in relation to HA [63].

We found that having pre-existing mental health conditions was the strongest determinant of HA and this aligns with existing pandemic research on broader anxiety and mental health outcomes. During the COVID-19 pandemic, individuals with pre-existing mental health conditions have been widely reported to experience greater mental health symptoms than those without, particularly in terms of anxiety [57, 64–67]. However, understanding this relationship is complicated by the substantial overlap between HA and other mental health conditions, such as panic disorder and obsessive-compulsive disorder (OCD) [55]. HA shares symptoms with panic disorder, such as hypervigilance and exhibits intrusive thoughts and repetitive behaviours characteristic of OCD [55]. Moreover, this intersection with OCD has prompted the conceptualisation of HA under OCD, presenting diagnostic challenges in clinical and research settings [55]. Therefore, further exploration of the complex interplay between HA and other mental health conditions is needed, particularly concerning preventative and intervention strategies.

In accordance with our findings, previous research has shown that pre-existing physical health conditions are associated with both elevated anxiety during the COVID-19 pandemic and pre-pandemic [56, 68]. These observations are unsurprising given that individuals with physical health conditions face a persistent internal threat from their symptoms, as described in the cognitive-behavioural model of HA [6–8]. In the context of the pandemic, where an external threat is presented, individuals with physical health conditions may experience heightened perception of illness vulnerability and in turn, HA [2, 15]. However, these findings warrant further investigation as greater comorbidity is typically associated with older age, which contrasts the higher HA levels observed in younger individuals in existing studies [17–19, 69].

Despite existing research suggesting younger age to be associated with greater anxiety outcomes, this relationship could only be explored narratively due to heterogeneity in data, revealing inconsistent findings [17–19]. Our analysis also indicated that psychological factors, such as feelings of hopelessness and intolerance of uncertainty are key determinants of HA. This suggests that public health agencies could help to reduce HA in pandemics by providing appropriate reassurance to reduce psychological factors that can exacerbate HA, such as uncertainty and hopelessness, and should be conscious of not causing unnecessary concern. It was interesting that greater consumption of media during the pandemic, such as COVID-19 news viewing, and social media use, were associated with HA. Utilising these media platforms to deliver more evidence-based information could help to balance information from less credible sources and potentially reduce HA. Additionally, we found poor community cohesion was related to HA, so improving this could help to reduce HA and have additional benefits such as improving vaccine uptake.

### Strengths and limitations

This systematic review and meta-analysis has several strengths. This review is the first of its kind to summarise existing evidence concerning the prevalence and determinants of HA during the COVID-19 pandemic. Additionally, all studies in this review were rated moderate to high quality, enhancing the reliability of evidence included in the analysis.

The limitations of this review were that most studies employed a cross-sectional design, limiting the ability to establish temporal relationships between HA and the COVID-19 pandemic. In addition, the few studies that used a cohort design displayed considerable heterogeneity in baseline and comparator sampling periods, preventing the establishment of longitudinal effects. Furthermore, all but one study used a convenience sampling method, with most surveying on social media platforms. Although the COVID-19 pandemic impeded alternative sampling methods due to safety concerns, the reliance on convenience sampling introduces issues, such as selection bias and limited external validity. Future research should employ rigorous sampling methods to ensure that samples are diverse and accurately reflect the population. The findings of this review indicated that most samples were non-representative of the population as samples were predominantly female, which could have led to an overestimation of the burden within the general adult population given that higher levels of anxiety are typically reported in females. There was also a lack of ethnicity data among included studies, which reduced the generalisability of the findings and hindered the exploration of ethnicity-related disparities. HA may differ across ethnic groups as ethnic minorities are more likely to experience mental health problems and often face barriers to accessing services, potentially leading to higher levels of HA.

This review was also limited as there was a large degree of heterogeneity in the studies reviewed, with how they measured key determinants of HA such as age and only individual studies measured some determinants. Therefore, it was not possible to conduct a meta-analysis for all determinants to provide an overview of their effect on HA. A further limitation to this review is that the search strategy may not have retrieved all the relevant papers due to the inclusion of English language publications only and it did not include grey literature. Finally, the CCAT form used to assess methodological quality is tailored to the evaluation of cross-sectional studies, which may overestimate the quality of included studies [70]. The exclusion of qualitative studies may also have limited our understanding of the determinants of HA and important contextual factors that influence HA.

### Future research

Future research should build upon the presented findings and explore longitudinal trends to understand the long-term psychological impact of COVID-19 in terms of HA. Researchers should aim to explore individual characteristics of HA through an itemised breakdown of the SHAI-18 to identify distinct features in subgroups and pandemic-specific HA. Furthermore, the efficacy of intervention strategies aimed at mitigating HA during public health crises, such as online Cognitive behavioural therapy (CBT), should be explored within a systematic review. Online CBT could be particularly helpful for treating HA according to the cognitive-behavioural model of HA and online CBT offers several benefits, including greater accessibility, convenience, cost-effectiveness, and flexibility compared to in-person therapy [8]. Future research should encompass diverse and representative samples to investigate potential risk factors, including those highlighted in this review and previous studies, to establish disparities in the prevalence and treatment of HA. In addition, more attention is needed to explore the intersection between HA and other mental health conditions to improve diagnostic accuracy and current treatment options.

## Conclusion

This systematic review and meta-analysis presents evidence suggesting elevated HA in the general adult population during the COVID-19 pandemic compared to pre-pandemic studies. This relationship appears to be variable, with females, unmarried individuals and those with pre-existing physical and mental health conditions exhibiting higher levels of HA. The recent pandemic has highlighted the importance of addressing HA in future public health crises. This underscores the urgent need for mental and public health strategies aiming to mitigate the psychological effects in future crises. Our review indicates that public health interventions could be particularly effective if they target individuals who are potentially more at risk of HA, such as females, unmarried individuals, and those with pre-existing physical and mental health conditions. Further research that is both representative and longitudinal is needed to establish temporal effects across groups. These findings will enhance existing understanding of the multifaceted nature of HA and inform effective prevention and treatment strategies applicable to public health crises for a wide range of anxiety-related conditions.

## Supporting information

S1 Text Example search strategy for MEDLINE. (DOCX)

S1 Table List of all studies identified in the search. (XLSX)

S2 Table Quality assessment of included studies. (DOCX)

S1 Checklist PRISMA 2020 checklist. (DOCX)
