# Supplementary material for: The prevalence and determinants of health anxiety during the covid-19 pandemic: A systematic review and meta-analysis
Source: PLOS Ment Health. 2024 Dec 30;1(7):e0000120. doi: 10.1371/journal.pmen.0000120 (PMC12798481; doi:10.1371/journal.pmen.0000120)
Supplement: S1 Text — (DOCX) [file pmen.0000120.s001.docx]

**S1 Text. Example search strategy for MEDLINE**

This strategy was adapted for PsychINFO, Embase and Web of Science Core Collection.

| **Health Anxiety** | **COVID-19** |
| --- | --- |
| health anxiet*.mp.  exp hypochondriasis/  hypochondri*.mp.  illness anxiet*.mp.  somatic symptom disorder.mp.  fear of illness.mp.  COVID-19 anxiet*.mp.  COVID anxiety  coronavirus anxiet*.mp.  pandemic anxiet*.mp.  disease anxiet*.mp.  medical anxiet*.mp. | exp COVID-19/  COVID-19.mp.  COVID.mp.  exp coronavirus/  coronavirus.mp.  exp SARS-CoV-2/  SARS-CoV-2.mp.  COVID-19 pandemic.mp.  COVID pandemic.mp.  coronavirus pandemic.mp.  SARS-CoV-2 pandemic.mp. |
