# Supplementary material for: The prevalence and determinants of health anxiety during the covid-19 pandemic: A systematic review and meta-analysis
Source: PLOS Ment Health. 2024 Dec 30;1(7):e0000120. doi: 10.1371/journal.pmen.0000120 (PMC12798481; doi:10.1371/journal.pmen.0000120)
Supplement: S2 Table — (DOCX) [file pmen.0000120.s003.docx]

**S2 Table: Quality Assessment of Included Studies**

Scoring: 0 (lowest) to 5 (highest). Quality: Low (≤20), moderate (<30) and high (≥30).

| **Study (n=12)** | **Preliminaries** | **Introduction** | **Design** | **Sampling** | **Data collection** | **Ethical matters** | **Results** | **Discussion** | **Total score** | | **Quality** |
| --- | --- | --- | --- | --- | --- | --- | --- | --- | --- | --- | --- |
|  |  |  |  |  |  |  |  |  | **/40** | **%** |  |
| Bredemeier et al.(40) | 5 | 4 | 3 | 4 | 4 | 4 | 4 | 5 | 33 | 82.50 | H |
| Chan et al.(41) | 4 | 4 | 3 | 3 | 3 | 4 | 4 | 4 | 29 | 72.50 | M |
|  |  |  |  |  |  |  |  |  |  |  |  |
| Heinen et al.(42) | 5 | 5 | 4 | 3 | 5 | 4 | 4 | 5 | 35 | 87.50 | H |
| Tull et al.(43) | 5 | 5 | 3 | 3 | 4 | 3 | 3 | 4 | 30 | 75.00 | H |
| Canli et al.(46) | 3 | 5 | 3 | 2 | 3 | 3 | 4 | 4 | 27 | 67.50 | M |
| Kirmizi et al.(47) | 4 | 4 | 4 | 4 | 4 | 4 | 4 | 4 | 32 | 80.00 | H |
| Kizilkurt et al.(51) | 4 | 5 | 4 | 4 | 4 | 4 | 4 | 5 | 34 | 85.00 | H |
| Ozdin et al.(52) | 4 | 5 | 4 | 5 | 4 | 4 | 4 | 4 | 34 | 85.00 | H |
|  |  |  |  |  |  |  |  |  |  |  |  |
| Svensson et al.(48) | 4 | 4 | 4 | 3 | 5 | 3 | 4 | 4 | 31 | 77.50 | H |
| Tull et al.(53) | 5 | 4 | 3 | 3 | 4 | 4 | 4 | 4 | 31 | 77.50 | H |
|  |  |  |  |  |  |  |  |  |  |  |  |
| Wechsler et al.(49) | 5 | 4 | 4 | 4 | 5 | 5 | 4 | 4 | 35 | 87.50 | H |
| Yalcin et al.(50) | 4 | 5 | 5 | 4 | 3 | 4 | 4 | 4 | 33 | 82.50 | H |
| **Mean** | 4.33 | 4.50 | 3.67 | 3.50 | 4.00 | 3.83 | 3.92 | 4.25 | 32.00 | 80.00 | - |
| **SD** | 0.65 | 0.52 | 0.65 | 0.80 | 0.74 | 0.58 | 0.29 | 0.45 | 2.49 | 6.22 | - |
